# Molecular Mechanisms of Injury in HIV-Associated Nephropathy

**DOI:** 10.3389/fmed.2018.00177

**Published:** 2018-06-07

**Authors:** Samuel J. Rednor, Michael J. Ross

**Affiliations:** ^1^Division of Nephrology, Albert Einstein College of Medicine/Montefiore Medical Center, Bronx, NY, United States; ^2^Department of Development and Molecular Biology, Albert Einstein College of Medicine, Bronx, NY, United States

**Keywords:** HIV, AIDS, HIV-associated nephropathy, proteinuria, chronic kidney disease, podocyte, renal tubular epithelial cell, APOL1

## Abstract

HIV-associated nephropathy (HIVAN) is an important cause of secondary focal glomerulosclerosis that occurs primarily in persons of African ancestry with advanced HIV disease. Although HIVAN is characterized by severe proteinuria and rapid progression to end stage renal disease without treatment, the phenotype is markedly attenuated by treatment with antiretroviral medications. HIV infection of glomerular and tubular epithelial cells and subsequent viral gene expression is a key contributor to HIVAN pathogenesis and the kidney can serve as reservoir for HIV strains that differ those in blood. HIV gene expression in renal epithelial cells leads to dysregulation of cellular pathways including cell cycle, inflammation, cell death, and cytoskeletal homeostasis. Polymorphisms in the APOL1 gene explain the marked predilection of HIVAN to occur in persons of African descent and HIVAN. Since HIVAN has the strongest association with APOL1 genotype of any of the APOL1-associated nephropathies, studies to determine the mechanisms by which HIV and APOL1 risk variants together promote kidney injury hold great promise to improve our understanding of the pathogenesis of APOL1-mediated kidney diseases.

## Epidemiology and clinical presentation of HIV-associated nephropathy

HIV-associated nephropathy (HIVAN), was first described early in the HIV epidemic in U.S. urban centers serving large numbers of HIV-positive persons of African descent. In the early 1990's, HIVAN was the most rapidly increasing cause of ESRD in the U.S., however, the widespread use of combination antiretroviral therapy (cART), in addition to markedly reducing the incidence of mortality and progression to AIDS, has resulted in a marked reduction in the incidence of classic HIVAN (1). While the incidence of ESRD attributed to HIVAN has declined since the introduction of cART, it has not dropped as dramatically as mortality or progression to AIDS (2). Since the phenotype of HIVAN is markedly attenuated by cART (1, 3), resulting in much lower levels of proteinuria and slower progression to severe CKD/ESRD (reducing likelihood of kidney biopsy), and the United States Renal Data System no longer collects data on HIV seropositive status, it is difficult to reliably estimate the current incidence/prevalence of HIVAN.

Patients with classic HIVAN present with rapidly progressive renal failure in conjunction with severe proteinuria and most have advanced HIV disease/AIDS with of CD4 counts <200 cells/mm^3^. These patients typically have enlarged hyperechoic kidneys on ultrasound with a bland urine sediment (4, 5). This classic presentation is rarely encountered in patients receiving cART. There are no serologic tests that accurately predict the presence of HIVAN and since HIV-positive patients are at increased risk for comorbidities that increase the risk of kidney disease, including diabetes mellitus and hepatitis C infection, HIVAN can only be definitively diagnosed by kidney biopsy.

There have been isolated case reports of HIVAN occurring in HIV-2 positive patients but the incidence of kidney disease in the setting of HIV-2 infection is unknown (6). Though the mechanism by which HIV-2 causes kidney disease has not been studied, since the HIV-2 genome encodes most of the same genes as HIV-1, it is plausible that the pathogenesis of HIVAN occurring in HIV-1 and HIV-2-positive patients is similar (7).

## Histopathology

**Figure 1:**
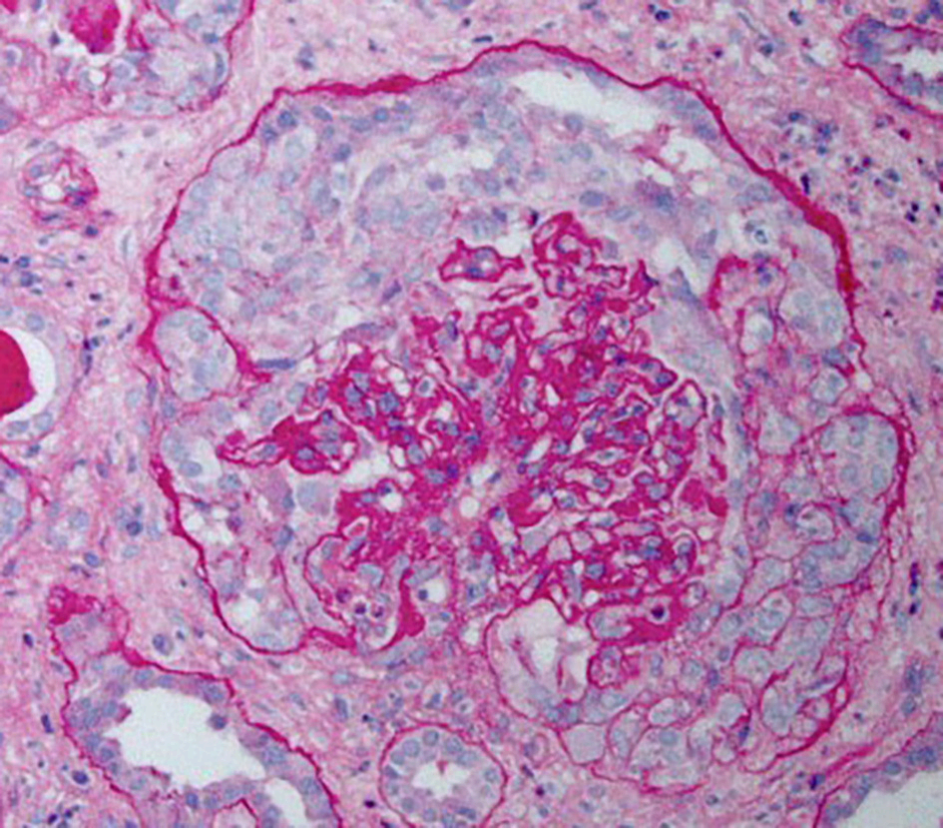
Periodic acid–Schiff stained kidney biopsy from a patient with HIVAN demonstrating collapsed glomerulus with focal global sclerosis and overlying pseudocrescent composed of proliferating glomerular epithelial cells. There is also prominent tubular atrophy and interstitial fibrosis.

HIVAN is characterized by the presence of FSGS and is most commonly associated with the collapsing variant (8). Proliferation and hypertrophy of overlying glomerular epithelial cells is often present, which can result in the presence of pseudocrescents [Figure 1, (9)].

Tubulointerstitial disease is an important component of the histopathology of HIVAN and may overshadow the severity of glomerular injury. Classic findings include dilation of tubular into “microcysts” (at least three times the diameter of normal adjacent tubules) and interstitial fibrosis and inflammation (8).

There are no specific immunofluorescence findings in HIVAN, however, as these patients often have increased plasma levels of immunoglobulins and coexisting infections, variable amounts of immunoglobulins and/or complement may be present and if prominent, may reflect the presence of a superimposed immune complex disease. Electron microscopy reveals podocyte foot process effacement, normal basement membrane thickness, absence of immune deposits, and presence of endothelial cell tubuloreticular inclusions, which likely reflect high circulating interferon levels present in patients with HIVAN (8).

## Infection of renal epithelial cells by HIV

HIV can infect renal tubular epithelial cells (RTEc), podocytes, and parietal epithelial cells and this infection is not specific to HIVAN but also occurs in patients with other forms of kidney disease, including HIV-positive patients with diabetic kidney disease (10, 11). Since renal epithelial cells do not normally express CD4, the primary receptor for HIV-1, or the co-receptors CCR5 and CXCR4 (12), the mechanisms by which infection occurs remain incompletely understood. Ray et al. reported that cell-free clinical HIV viral isolates were able to infect human RTEc via a CD4-independent mechanism and that HIV-infected mononuclear cells were able to mediate direct cell-cell transfer of HIV to RTEc (11).

More recently, investigators demonstrated that transfer of HIV directly from infected lymphocytes to RTEc was markedly more efficient than infection by cell free virus and that cell-cell viral transfer did not require expression of CD4 on target cells or the HIV envelope protein (Env) and was mediated in part, by heparan sulfate proteoglycans (13). Importantly, further studies showed that infected RTEc can also transfer HIV to uninfected primary T lymphocytes, suggesting that the infected RTEc may serve as a viral reservoir capable of infecting lymphocytes (14). These observations have important implications beyond the pathogenesis of HIVAN as they demonstrate that therapies aimed at fully curing HIV infection will need to include strategies to eliminate HIV infection in the kidneys.

Infection of renal epithelial cells is also an important issue in the context of kidney transplantation as a recent study involving 19 HIV-positive patients who received HIV-negative kidney allografts, found that 13 allografts had detectable HIV RNA in podocytes and/or RTEc, despite having undetectable plasma viral RNA. Moreover, those with infected kidneys had greater podocyte and RTEc injury and more rapid loss of kidney function (15).

A recent study elucidated a novel mechanism by which HIV-1 infects human podocytes (16). Podocytes cultured from children with HIVAN supported low level productive infection when exposed to cell free virus. The ability of cell-free HIV-1 to infect these podocytes was dependent upon the presence of the HIV envelope (*env*) gene and cell surface proteoglycans. Expression of transmembrane TNFα promoted HIV infection and subsequent integration into genomic DNA. The role of transfer of HIV-1 from infected mononuclear cells to podocytes was not investigated in this study.

The renal epithelium may also harbor viral strains that differ from those in blood from the same patient. In one study, investigators used laser capture microdissection to isolate DNA from RTEc in kidney biopsies from patients with HIVAN. Analysis of HIV sequences amplified from this DNA demonstrated diversity in the viral *env* sequences, suggesting ongoing replication and evolution of the virus. Further, comparison of kidney-derived sequences to those amplified from blood revealed that kidney and blood-derived viral sequences clustered separately, suggesting that the renal epithelium is a separate viral compartment that may harbor unique viral quasispecies (17). Recent work from Blasi, et al demonstrated similar findings using urine specimens. They found that 12 of 24 patients with HIV RNA detectable in plasma also had virus in their urine. Analysis of viral *env* sequences from blood and urine revealed that urine-derived sequences clustered separately from blood-derived sequences (18).

Studies in macaques demonstrated that the ability of chimeric simian-human immunodeficiency (SHIV) viral clones to cause glomerular and tubular injury varied significantly, strongly suggesting that viral sequence variation is an important determinant of kidney disease (19). It is not known whether patients' kidneys harbor quasispecies with distinct variations in HIV genes that mediate renal injury or alter response to cART. Whether glomerular epithelial cells can serve as viral reservoirs also remains to be determined.

## Pathomechanisms of HIVAN

### Role for HIV genes in causing HIVAN

**Figure 2:**
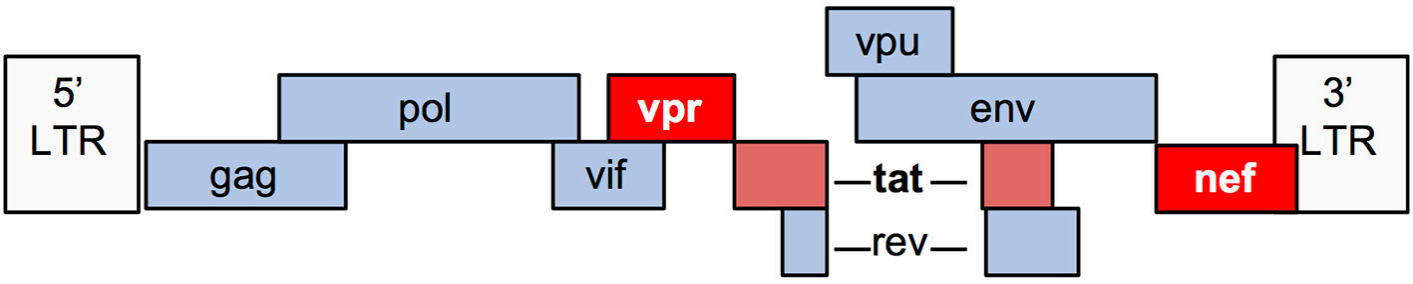
Schematic diagram of the HIV-1 genome.

The HIV-1 genome encodes 9 genes (Figure 2). Numerous *in vitro* and animal models have been used to study the mechanisms by which viral infection of renal epithelial cells can lead to HIVAN. The most commonly used model (“Tg26”) is transgenic for an HIV provirus lacking the *gag* and *pol* genes. These mice develop severe proteinuria, progressive kidney failure, and histologic kidney injury that closely models HIVAN (20). Since *gag* and *pol* encode the major structural and enzymatic viral proteins, these mice do not produce virus, thereby demonstrating that viral replication is not necessary for the HIVAN phenotype. Numerous transgenic rodent models have been generated, expressing various HIV genes using different promoters and together, these studies demonstrate that expression of *nef* and/or *vpr* is sufficient to generate the full HIVAN phenotype and the remaining genes are not necessary for the HIVAN phenotype in rodents (21).

Nef is a 27–34 kD myristoylated protein with important roles in the HIV lifecycle. Nef promotes viral transcription and activation of T cells, while helping infected cells to avoid immune surveillance by decreasing cell surface expression of several receptors including CD4, CXCR4, CCR5, and major histocompatibility complex class I (MHC-I) (22). Nef also has myriad effects upon cellular signaling, including activation of Src family kinases (23).

Vpr is a 14 kD protein that is important for nuclear import of HIV preintegration complexes. Vpr also has several other important effects upon infected cells, including inducing cell cycle arrest in G2/M phase and is an important mediator of HIV-induced injury and death (24).

Tat is critical for transactivation of HIV transcription in human cells, but is less active in murine cells due to lack of cyclin T1 in the mouse genome (25), which may explain why Tat does not have an important role in murine HIVAN models (26). However, *in vitro* studies using human cells suggest that Tat may contribute to glomerular injury in HIVAN, in part, via its ability to upregulate proinflammatory cytokines (27, 28).

### Mechanisms of glomerular injury

#### Cell cycle dysregulation and dedifferentiation

During the course of glomerular development, podocytes undergo proliferation and maturation through exquisitely controlled developmental programs, resulting in mature podocytes, which are terminally differentiated and unable to proliferate (29). Cell cycle dysregulation and aberrant podocyte cell cycle reentry is a hallmark of HIVAN pathogenesis. It has long been appreciated that in HIVAN, the proliferation marker Ki67 is expressed in podocytes overlying glomerular capillaries as well as in cells comprising the pseudocrescents surrounding the glomerular tufts in HIVAN biopsies and HIV-transgenic mice (9, 30). Though most early work in the field identified these cells as podocytes, more recent studies suggest that some or all cells comprising pseudocrescents in HIVAN and non-HIVAN collapsing FSGS may be parietal epithelial cells (PECs) (31). These discrepant findings may be explained by subsequent studies demonstrating that parietal epithelial cells may express podocyte genes at low levels (32) and studies in mice showing that PECs can be recruited onto glomerular tufts in the setting of glomerular injury (33, 34).

The cyclin dependent kinase (CDK) inhibitors p27 and p57 are highly expressed in podocytes and help to maintain them in a quiescent state by inhibiting activation of CDKs (35). p27 and p57 are down regulated in podocytes in HIVAN biopsies, thereby permitting CDK activation and entry into the cell cycle. In addition, Nef induces activation of Src family kinases in podocytes, which subsequently leads to activation of Stat3 and MAPK-induced proliferation and dedifferentiation (36). Blocking these pathways prevents HIV-induced podocyte proliferation and/or dedifferentiation and restores expression of key podocyte proteins including synaptopodin and WT-1 (36–38). Notch signaling pathways are also activated in HIVAN and inhibition of Notch signaling using a gamma secretase inhibitor prevents podocyte proliferation induced by Nef and Tat *in vitro* (39). Other signaling pathways that have been implicated in the pathogenesis of podocyte proliferation and/or dedifferentiation in HIVAN include retinoic acid receptor signaling (40) mammalian target of rapamycin (mTOR) (41) and Kruppel-like factors (42, 43).

Podocyte function is highly dependent upon maintenance of a complex and dynamic cytoskeleton. In HIVAN, there is widespread foot process effacement, which is due, in part to cytoskeletal dysregulation. Activation of Src kinase by HIV gene expression leads to activation of Rac1 and inhibition of RhoA, which results in loss of actin stress fiber formation (44). HIV Tat has recently been shown to promote fibroblast growth factor-2 mediated dysregulation of MAPK and RhoA in human podocytes *in vitro* (28). Moreover, HIV gene expression also reduces expression of podocyte genes, such as synaptopodin, that are important mediators of cytoskeletal integrity (45, 46).

#### Cellular injury and death

Several studies have delineated mechanisms of HIV-induced podocyte death. Since podocytes are terminally differentiated cells, aberrant reentry of podocytes into the cell cycle can lead to mitotic catastrophe (47). Nef expression in HIV infected glomerular epithelial cells may induce death of podocytes via this mechanism while inducing proliferation of parietal epithelial cells in an attempt to replace podocytes. Many podocyte genes are dysregulated by HIV infection *in vitro* and in animal models and genes with likely roles in HIV-induced podocyte death include *APOL1* (discussed below) (48–50), Fas (51), KLF6 (52), p53 (53), and p66ShcA (54), the NLRP3 inflammasome (55).

In the pre-cART era, small clinical studies suggested that treatment with ACE inhibitors improved renal outcomes in HIVAN (56, 57). Several studies have also demonstrated that ACE inhibitors and angiotensin receptor blockers (ARBs) also prevent renal injury in HIV transgenic mice (58, 59). Interestingly, the protective effects of ARBs in HIV-transgenic mice appear to be independent of blockade of angiotensin II receptor type 1 on podocytes (60).

**Figure 3:**
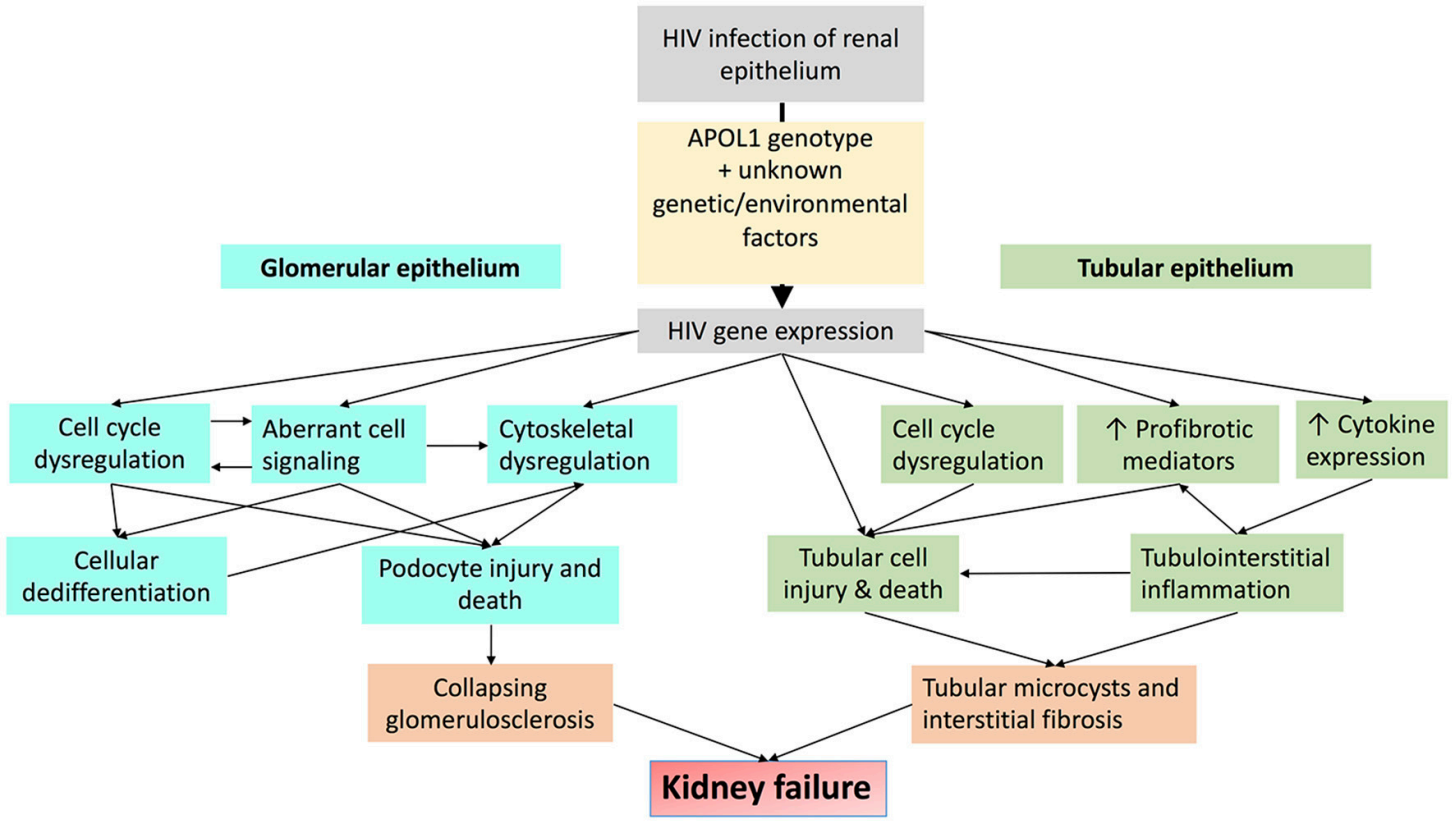
Schematic diagram demonstrating major mechanisms by which HIV-1 infection promotes glomerular and tubular injury.

Dysregulated cell-cell and cell-matrix adhesion also contribute to glomerular injury in HIVAN. Sidekick-1 (Sdk-1) is a transmembrane immunoglobulin family protein that mediates cell-cell adhesion. HIV expression increases Sdk-1 expression which is primarily detected in glomerular pseudocrescents HIV-transgenic mice. Sdk-1 mediated dysregulation of cell-cell adhesion may contribute to the clustering of glomerular epithelial cells that is characteristic of collapsing glomerulopathy in HIVAN (61, 62). HIV expression also suppresses activation of the small GTPase RAP1 by increasing expression of RAP1GAP, which is a negative regulator of RAP1. RAP1 is an important regulator of cell-cell and cell-matrix adhesion. Reduced RAP1 expression reduces β1-integrin abundance, leading to podocyte detachment and glomerulosclerosis in mice (63). Major mechanisms of HIV-induced glomerular injury are summarized in Figure 3.

### Tubulointerstitial injury in HIVAN

#### Cell cycle dysregulation

Though Ki-67 is also upregulated in RTEc in HIVAN biopsies, it is not clear whether this is a primary pathogenic process or is an adaptive response to replace RTEc undergoing apoptosis. Also, though Ki-67 is commonly used as a marker of proliferation, it is expressed during all phases of cell cycle other than G_0_ (64). Since Vpr causes G2/M phase arrest and dysregulated cytokinesis in RTEc *in vitro* and in mice (65, 66), it is possible that the increased Ki-67 in RTEc in HIVAN reflects increased proportion of cells that arrested in cell cycle phases other than G_0_ and not ongoing proliferation *per se*. Moreover, since G2/M phase arrest of RTEc has recently been demonstrated to be an important mechanism contributing to tubulointerstitial injury and fibrosis in non-HIV kidney injury in mice (67), it is likely that mechanisms of cell cycle dysregulation in HIVAN may have direct relevance to our understanding of the tubulointerstitial injury in other proteinuric kidney diseases.

#### Cellular injury and death

Apoptosis is increased in HIVAN biopsies, with the greatest levels of apoptosis found in RTEc (68, 69). Vpr expression induces apoptosis in RTEc *in vitro*, which is mediated via persistent unregulated activation of ERK, resulting in caspase 8 activation, which cleaves BID to tBID, leading to mitochondrial membrane permeablization and apoptosis (65). Mitochondrial injury and Vpr-induced apoptosis are dependent upon expression of the ubiquitin-like protein FAT10 (70). Interestingly, suppression of FAT10 expression prevents apoptosis primarily in cells with Vpr-induced hyperploidy *in vitro*.

Homeo-domain interacting protein kinase 2 (HIPK2) has also recently been implicated in the pathogenesis of HIV-induced RTEc apoptosis and tubulointerstitial fibrosis. HIPK2 expression is increased, primarily in tubular cells in HIV-transgenic mice and in HIVAN biopsy specimens and promotes RTEc apoptosis via activation of p53, TGF-β-SMAD3, and Wnt/Notch pathways which together promote tubulointerstitial fibrosis (71).

#### Inflammation

The predominant transcriptional response of RTEc after HIV infection is increased expression of proinflammatory mediators, many of which are NF-κB and interferon-inducible genes (72, 73). A primary role for NF-κB-induced inflammation in the pathogenesis of HIVAN is supported by studies demonstrating that pharmacologic NF-κB inhibition prevents renal disease in two different murine models of HIV-induced kidney disease (74, 75). Further, clinical studies (primarily from the pre-cART era) demonstrated that glucocorticoid treatment is associated with improved renal outcomes in HIVAN (76–78) and that the primary histologic change observed after treatment is reduced tubulointerstitial inflammation (79).

Treatment of HIV-transgenic mice with the mTOR inhibitor sirolimus is also protective against the HIVAN phenotype in HIV-transgenic mice. Sirolimus was shown to reduce HIV transcription in podocytes *in vitro* and in mice (80) and prevent expression of mediators of epithelial mesenchymal transition (81) in these models. Since mTOR inhibition can have myriad cellular effects, including immunosuppression and activation of autophagy (a cytoprotective and immunomodulatory pathway), it is likely that many pathways are involved in mTOR-induced protection in murine models of HIVAN.

Major mechanisms of HIV-induced tubular injury are summarized in Figure 3.

### Role of apoliproprotein L1 variants in HIVAN

#### Epidemiology

**Figure 4:**
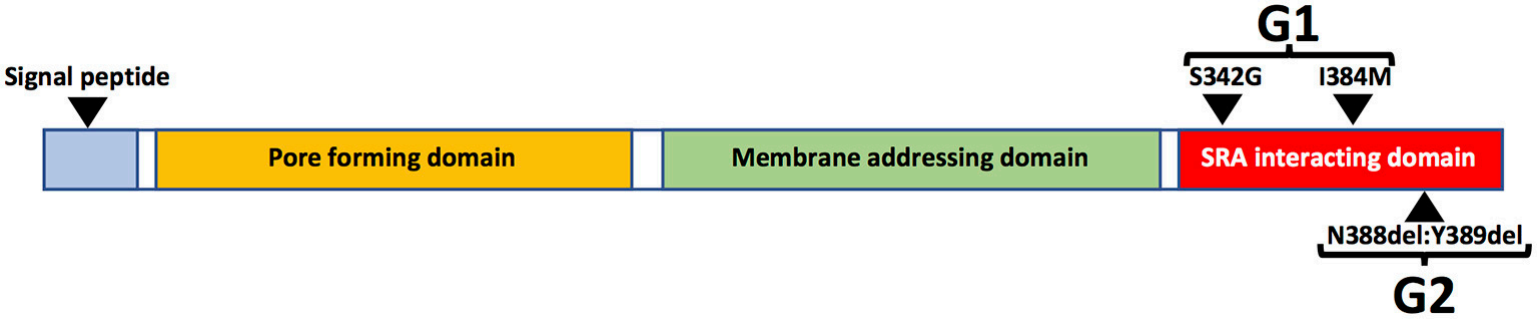
Schematic diagram of major APOL1 protein domains and G1 and G2 sequence variants.

The risk of HIVAN in persons of African ancestry is much higher than in other racial/ethnic groups and black race is associated with a 12.2-fold increased risk of HIVAN (82). The genetic basis of this disparity was elucidated in a seminal study in 2010 in which the authors reported that variants of the *APOL1* gene, which encodes the Apolipoprotein L1 (ApoL1) protein, are strongly associated with risk of kidney disease. Two variants (G1 and G2) are associated with increased risk of kidney disease compared to wild type (G0). The G1 allele encodes two missense mutations in the APOL1 protein whereas G2 encodes a two-amino acid deletion (Figure 4). Kidney risk attributed to the G1 and G2 alleles generally occurs in an autosomal recessive pattern and persons who are homozygous for either allele or compound heterozygous for G1 and G2 are at approximately equally increased risk of kidney disease (83).

Persons with APOL1 high risk genotypes are at 10.5- and 7.3-fold increased risk of developing non-HIV related FSGS and hypertension-attributed ESRD, respectively (83, 84). Remarkably, subsequent studies found that APOL1 high risk genotypes are associated with 29-fold increased risk of HIVAN in African Americans and an 89-fold increase risk in South Africans and HIV-positive patients are at 50% lifetime risk of developing HIVAN without antiretroviral treatment (85, 86). Importantly, though nearly all studies have demonstrated that heterozygosity for APOL1 risk alleles does not increase the risk of kidney disease, Kasembeli et al reported that in HIV positive South Africans, heterozygosity for the G1, but not the G2 allele, was associated with increased risk of HIVAN (86). Though this observation requires replication in another cohort, if true, it may be due to differences in genetic admixture of the South African population or environmental factors.

The APOL1 G1 and G2 alleles are unique to persons of African ancestry, which is explained by the fact that these alleles arose relatively recently in Africa. Even within Africa, the prevalence of these alleles varies widely—the prevalence of the G1 allele is highest in western African countries including Ghana and Nigeria where the prevalence of the allele is >40%, but the prevalence of the G2 allele is more variable at 6–24% (87). In the United States, the prevalence of the G1 and G2 alleles in African Americans is 20–22 and 13–15% respectively, and 10–15% have high risk APOL1 genotypes. Due to the transatlantic slave trade that occurred from Western Africa the sixteenth to the nineteenth centuries, the APOL1 risk alleles are also found among persons living in the Caribbean and Latin America at highly variable frequencies (88).

#### Putative mechanisms of APOL1-mediated kidney injury

The mechanisms by which APOL1 variants promote killing of trypanosomes may provide important insights into how they promote renal injury. APOL1 functions in part, as an innate immune response protein, and is induced by interferons and TNF-α (89). APOL1 is unique to a few primate species and is not encoded in the genomes of non-primate species. *Trypanosoma brucei rhodesiense* causes African Sleeping Sickness in humans who are homozygous for the APOL1 G0 allele because it produces Serum Resistance Associated (SRA) protein, which binds APOL1, preventing it from killing the parasite. The changes in APOL1 encoded by the G1 and G2 alleles prevent the SRA protein from binding/inactivating APOL1, thereby protecting persons harboring one or both of these alleles from African Sleeping Sickness induced by *Trypanosoma brucei rhodesiense* (90).

APOL1 can be expressed in numerous cell types and tissues, especially in the presence of interferons and/or TNFα (89). There are also several isoforms of APOL1, most of which contain an N-terminal signal peptide, which is likely necessary for extracellular secretion. The liver is the source of most circulating APOL1 protein, which is complexed in HDL3 particles, which are also synthesized in liver (91). There are also APOL1 isoforms that lack the N-terminal signal peptide sequence (Figure 4) and are expressed as intracellular proteins (49). Plasma APOL1 levels do not correlate with risk of kidney disease (92, 93). Moreover, in the setting of kidney transplantation, the presence of high risk APOL1 genotypes in the kidney donor, but not the recipient, is strongly associated with adverse allograft outcomes (94–96). These data strongly suggest that local production of APOL1 in kidney cells is more important than circulating APOL1 in promoting kidney injury.

**Figure 5:**
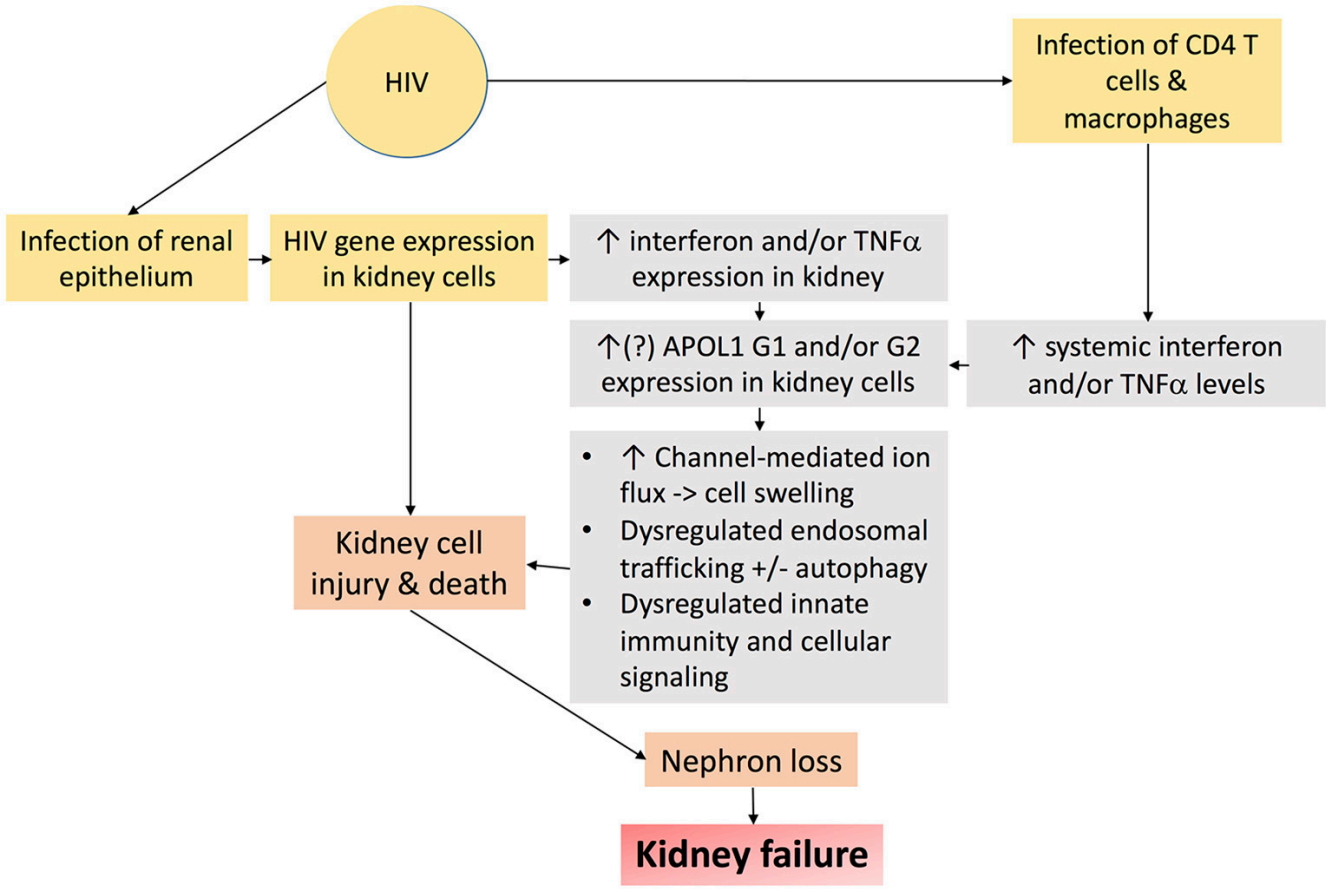
Schematic diagram demonstrating putative mechanisms by which HIV-1 infection and APOL1 together promote kidney injury.

Nearly 15% of African Americans have high risk genotypes, the majority of whom never develop kidney disease. Additional factors or “second hits” are therefore necessary to unmask the deleterious effects of APOL1 upon the kidney. Several of these second hits have been identified, including exogenous interferon administration (89), lupus nephritis (97, 98), and sickle cell disease (99), but HIV is the most potent “second hit” to promote kidney disease in persons with high risk APOL1 genotypes (85, 86). HIVAN is therefore an important model disease, the study of which may hold important insights mechanisms by which APOL1 promotes kidney disease. Putative mechanisms by which APOL1 and HIV-1 may together promote kidney injury are depicted in Figure 5.

Though several types of kidney cells have been reported to produce APOL1 *in vivo*, including podocytes, tubular epithelial cells, endothelial cells, and vascular smooth muscle cells (100, 101), it remains unclear which cells are the most important targets of APOL1-induced injury. Moreover, while cytokines strongly induce APOL1 expression *in vitro* and many of these same cytokines are commonly increased in the context of chronic kidney injury, APOL1 expression in biopsy specimens from patients with HIVAN has been reported to be lower than control specimens. Though it is possible, if not likely that APOL1 expression is increased at some point in the course of the disease process, it is not clear whether increased expression of APOL1 is an important contributor to disease pathogenesis. This point also has important implications with regard to relevance of current *in vitro* and animal models of APOL1-mediated injury, most of which rely on overexpression of APOL1 at non-physiologic levels or expression of APOL1 transgenes in animals that normally lack APOL1 in their genomes.

Numerous publications have elucidated potential mechanisms by which APOL1 risk alleles may promote kidney injury but relatively few have focused upon APOL1-mediated injury in the context of HIV infection. APOL1 kills trypanosomes by inserting into acidified endosomes, which when recycled to the plasma membrane at neutral pH become high conductance cation channels, resulting in rapid swelling and lysis of the parasite (102). Forced overexpression of APOL1 G1 and G2 alleles induce greater cytotoxicity than the G0 allele *in vitro* (49, 50, 89) but it remains unclear whether APOL1 promotes kidney injury via its function as a channel. Transgenic mice expressing the G0 or G2 alleles of APOL1 in podocytes under control of the nephrin promoter do not develop kidney disease (103) but mice expressing the G2 but not the G0 allele in podocytes under control of the podocin promoter developed FSGS lesions (48). Podocyte injury induced by the G2 allele in the latter study was associated with impaired endosomal trafficking and autophagic flux, leading to pyroptosis (48). *In vitro* studies with human podocytes found that HIV infection markedly increased the toxicity of APOL1 overexpression (50).

The results of a recent provocative study suggest that APOL1-induced cytotoxicity and channel function may be artifacts of *in vitro* overexpression and that these effects may not occur when APOL1 is expressed at physiologic levels (104). Since this study used inducible APOL1 in immortalized cell lines, additional studies are needed to determine if this observation is relevant in primary kidney cells. However, it is clear that since nearly all previous studies on the role of APOL1 in inducing cellular injury used overexpression models, further studies are needed in which APOL1 is expressed at physiologically relevant levels in physiologically relevant cells.

#### Future perspectives

Though antiretroviral medications have emerged as an effective strategy for the prevention and treatment of kidney disease in HIV-positive persons, there remains an urgent need for research to answer key questions with important implications for the care for all persons living with HIV and/or kidney disease including: (1) How do antiretroviral medications prevent/treat HIVAN without reducing HIV infection of renal epithelial cells? (2) Does the kidney harbor viral strains with unique antiretroviral resistance patterns? (3) Is it possible to eradicate HIV-1 from the kidney without worsening kidney injury by killing infected epithelial cells? (4) How do APOL1 risk variants promote renal injury and why is HIV-1 such a potent “second hit” in persons with APOL1 high risk genotypes? (5) Since HIVAN is the only “APOL1 nephropathy” with an effective treatment available (antiretrovirals), can insights into the pathogenesis of HIVAN inform treatment of APOL1-associated kidney disease in HIV-negative patients? (6) How can we develop *in vitro* and animal models that faithfully model the *in vivo* function(s) of APOL1?

## Conclusions

HIVAN is caused by infection of renal epithelial cells in genetically susceptible persons. In addition to its importance in HIVAN pathogenesis, the infected renal epithelia can serve as a viral reservoir that must be addressed in future attempts to cure HIV-positive patients. HIV-infection induces dysregulation of host genes and several cellular pathways, including inflammation, cell cycle, cytoskeletal homeostasis, and cell death. Polymorphisms in the *APOL1* gene account for the majority of excess risk of HIVAN attributed to African ancestry and of all APOL1-associated nephropathies, HIVAN has the strongest association with APOL1 risk genotypes. Though antiretroviral therapies have markedly reduced the burden of HIVAN in HIV-positive patients, studies to elucidate how HIV increases susceptibility to kidney disease in persons with high risk APOL1 genotypes promise to provide key insights that may help develop novel strategies for the prevention and treatment of APOL1 nephropathies in HIV-negative patients.

## Author contributions

SR prepared the initial draft of this manuscript. MR revised and expanded the scope of this manuscript.

### Conflict of interest statement

The authors declare that the research was conducted in the absence of any commercial or financial relationships that could be construed as a potential conflict of interest.
